# A Review of Health-Beneficial Properties of Oats

**DOI:** 10.3390/foods10112591

**Published:** 2021-10-26

**Authors:** Devendra Paudel, Bandana Dhungana, Melanie Caffe, Padmanaban Krishnan

**Affiliations:** 1 Dairy and Food Science Department, South Dakota State University, Brookings, SD 57007, USA; devendra.paudel@jacks.sdstate.edu; 2 Department of Agronomy, Horticulture and Plant Science, South Dakota State University, Brookings, SD 57007, USA; bandana.dhungana@jacks.sdstate.edu (B.D.); melanie.caffe@sdstate.edu (M.C.)

**Keywords:** oats, beta-glucan, cardiovascular disease (CVD), type-2 diabetes, obesity, cancer

## Abstract

Oat is among the food crops and ancient grains cultivated and consumed worldwide. It is gaining in popularity owing to its nutritional composition and multifunctional benefits of select bioactive compounds. Beta-glucan is an important component of dietary fiber found in oat grains. It is the major active compound in oats with proven cholesterol-lowering and antidiabetic effects. Oats also provide substantial levels of other bioactive compounds such as phenolic acids, tocols, sterols, avenacosides, and avenanthramides. The consumption of oats has been determined to be beneficial for human health by promoting immunomodulation and improving gut microbiota. In addition, oat consumption assists in preventing diseases such as atherosclerosis, dermatitis, and some forms of cancer. While much has been published in relation to oat nutrients and oat fibers and their impact on major diseases, the oat industries and consumers may benefit from greater knowledge and understanding of clinical effects, range of occurrence, distribution, therapeutic doses and food functional attributes of other oat bioactives such as avenanthramides and saponins as well as other anti-inflammatory agents found in the cereal. This review focuses on the various studies relevant to the contribution of the consumption of oats and oat-based products in preventing human diseases and promoting human health.

## 1. Introduction

Oat (*Avena sativa* L.) is unique among all cereal crops because it possesses many nutrients that bear value for human food, animal feed, health care, and cosmetics [1,2]. It is an annual crop cultivated for more than 2000 years in different parts of the world [3] and is one of the oldest crops known to human civilization [4]. It appeared in cultivation several thousand years later than other grains such as wheat and barley [5]. This cereal is an important source of carbohydrates, dietary soluble fiber, balanced protein, lipids, different phenolic compounds, vitamins, and minerals [6]. Because of the growing awareness of the public toward healthy eating habits, oat has received increased attention from scientific researchers and industries. Food-based companies are considering better nutritional composition together with the popularity of ancient grains and are developing novel food products by incorporating oats as an ancient grain in breakfast cereals, beverages, bread, and infant foods [7]. Although oats are mainly used in breakfast cereals and snack bars, the inclusion of it in different products would greatly benefit consumers because of its health promoting attributes [3,8]. Oat beta-glucan (OBG), one of the major components of soluble fiber, is a viscous polysaccharide made of a linear branched chain of D-glucose monosaccharides bonded by mixed β (1 → 3) and β (1 → 4) linkages. It is located in the endosperm cell wall of the kernel [9]. It is considered to be the major active component in oats with various functional and nutritional properties, mainly cholesterol lowering [10] and antidiabetic effects [11]. OBG content has been reported to range from 1.8 to 7% [12]. OBG content varies greatly among oat cultivars and is affected by growing locations, storage, and processing conditions [13]. Protein content in oat groats ranges from 13 to 20% [14]. Proteins are mostly present in the embryo (about 30%) [9]. Other minor components in oats are antioxidant compounds such as tocols, phenolic compounds, and sterols, which are also associated with health beneficial properties. Vitamin E activity is mainly contributed by tocopherols and tocotrienols, which together make tocols [15].

Several phenolic compounds such as avenanthramides (AVAs), p-hydroxybenoic acid, vanillic acid, tricin, ferulic acid, caffeic acid, protocatechuic acid, syringic acid, p-coumaric acid, sinapic acid, tricin, apigenin, luteolin, kaempferol, and quercetin have been identified in oats. Phenolic compounds are made of aromatic rings with one or more hydroxyl groups and are the products of secondary metabolism [16]. These phenolic compounds act as a defense mechanism against various pathogens, and their consumption is associated with the prevention of diseases like cancer, stroke, and coronary heart diseases [17]. The phenolic acid content on oat bran concentrate was found to be higher than in other oat products—oat bran, flaked oats, rolled oats, and oatcake—as the majority of phenolic compounds are located in the barn layer of the oat grain [18]. Ferulic acid (58–78%), followed by caffic acid and sinapic acid, are the most abundant phenolic compounds available in oat products. The free-radical scavenging ability and high antioxidant activity of these moieties make oats a unique promoter of health [18].

Sterols and phytic acid prevent the production of metal-mediated free radicals [11]. AVAs are phenolic alkaloids found exclusively in oats. AVAs have been documented as antioxidant, anti-inflammatory, antiproliferative, and anti-itching [19]. The most abundant AVAs found in oats are 2c, 2f, and 2p, although 25 different types of AVAs have been detected in oats. Oats also contain another unique phytochemical called steroidal saponin, mainly avenacins and avenacosides. Apart from plant defense mechanisms, oat saponins have a potential for cholesterol lowering, immunoregulatory, and anticancer activities [3].

Saponins are found to be effective against the growth of colon cancer cells [20], and their content in rolled oats and oat porridge was reported as 0.9 g/kg and 0.1 g/kg (as consumed), respectively [21]. However, detailed research about the level of saponin content in oat or oat products, their chemical composition, and their associated health benefits has yet to be conducted [20].

In this review, we summarize the status of the scientific knowledge and potential health beneficial effects of oats and its bioactive components. Table 1 is the compilation of the results of the research work done on the health benefits of oats.

### 1.1. Oats for Cardiovascular Diseases

Cardiovascular diseases (CVD) are among the leading causes of human deaths worldwide, and a study has shown that dietary risk is the major contributor for CVD. Based on National Health Interview Survey data, in 2017, 485.6 million people were under the prevalence of CVD worldwide, and this number increased by 28.5% in 10 years [105]. Mathers and Loncar (2006) [106] projected that CVD would claim 23.3 million people’s lives by 2030.

High levels of serum cholesterol and low-density lipoproteins (LDL) are known to increase the risk of CVD. The consumption of oats, however, was shown to reduce serum total cholesterol and LDL cholesterol, thereby reducing the risks of CVD [10,43,107,108,109]. A dietary approach, has been recommended as one of the most practical approaches for the treatment of LDL cholesterol [1,110]. Studies show that beta-glucan-rich oats or oat-based products in a diet significantly decrease the blood lipid profile and blood pressure by regulating insulin metabolism in a mild hypercholesterolemia subject [22,23,24,32,36]. Viscous OBG has been attributed to slowing the absorption of macronutrients in the digestive tract, which lowers postprandial blood glucose and the insulin response, thereby reducing blood pressure [111,112]. A dose-response relationship study in a mouse model showed that a higher amount of OBG is accompanied by a higher reduction of serum cholesterol level [25]. However, in one of the studies where healthy young men were fed with oat gum containing 9 g OBG daily for 14 days, there was no significant difference in the reduction of total serum cholesterol, LDL cholesterol, HDL cholesterol, and triglyceride concentrations between test group or control group. The authors suggested that the cholesterol-lowering property of oat bran should be measured not by the beta-glucan content but by the solubility and viscosity of OBG [35]. A study done on hypercholesterolemic subjects also supports this conclusion [113]. The amount and molecular weight (MW) of OBG that solubilizes in the intestine together with other compounds present in an oat product and their association also determine the cholesterol-lowering property [114,115].

Unrefined and whole oat-based products are more effective in lowering cholesterol as compared to processed oat products where oat tissues are highly disrupted. Studies suggest that for a similar amount of OBG, liquid-based foods are more effective than solid or semi-solid food. Hydrothermal processing is found to increase the extractability and reduce the solubility of OBG. Extracted OBG could also be depolymerized when incorporated into the food products [115]. β-glucanase contained in wheat also deactivates OBG in bakery products containing wheat during the fermentation process [114].

The cholesterol-lowering property of OBG is mainly mediated through the formation of a viscous layer in the small intestine, thereby inhibiting the uptake of cholesterol and increasing the excretion of bile acids by preventing its reabsorption. The inhibition of bile acid reabsorption stimulates the synthesis of bile acids from the available cholesterol, which in turn reduces the circulating LDL cholesterol [1,30,116].

It has also been proposed that soluble fiber helps in the reduction of cholesterol synthesis through an alteration in serum concentrations of hormone or short-chain fatty acids (SCFA)—acetate, propionate, and butyrate—which ultimately affects lipid metabolism. SCFA butyrate concentration significantly decreases the de novo synthesis of cholesterol in isolated rat hepatocytes [33]. Another study reported an increase of serum acetate and propionate levels following the consumption of oat bran diet, and the authors suggested that the SCFA response might have contributed to the cholesterol lowering effect [37].

Because of the number of studies and evidence supporting the beneficial roles of OBG, the United States Food and Drug Administration (FDA) approved the use of health claims on oat-based products, contributing to lowering the risk of CVD if consumed at the rate of 3 g per day of beta-glucan and if the food product contributes 0.75 g of beta-glucan per serving [117]. Similarly, Health Canada, the European Food Safety Authority, Food Standards in Australia and New Zealand, and the Ministry of Health in Malaysia have also approved health claims for oats related to the association between OBG and cholesterol [10].

In addition to beta-glucan, proteins and lipids in oats also contribute to lowering cholesterol [26,27]. Oat are rich in protein, ranging between 12 to 20% of oat groats. Oat proteins are high in albumins and globulins and low in prolamins. Prolamins have a lower lysine content than albumins and globulins [118], and hence oat protein has a higher biological value than other cereals with high prolamin content, and the Limited Amino acid (LAA) score of oat flour is 66.9, whereas wheat flour’s is 49.8 [119]. Oat protein may help to decrease the serum total and LDL cholesterol levels because they have low Lysine/Arginine and Methionine/Glycine ratios [26]. Favoring the excretion of fecal steroid, which would lead to a hepatic conversion of cholesterol into bile acid and the elevation in the expression of the LDL receptor, are possible mechanisms behind the cholesterol-lowering property of oat protein [120]. Oat is a good source of dietary fats, as the lipid content in oat groats ranges from 5 to 9%, which is the highest among cereal grains [121]. Tong et al. (2014) [31] reported that the consumption of oat oil by rats promoted the excretion of fecal lipids and bile acids. However, more evidence is required to confirm the cholesterol-lowering property of oat oil, as not all studies are in agreement [122].

### 1.2. Oats for Type II Diabetes

Type-2 diabetes is the most common metabolic disease in the world. International Diabetes Federation (IDF) reported that about 451 million people (age 18–99) had diabetes in 2017. This number is expected to reach 693 million by 2045 [123]. According to the National Diabetes Statistics Report, about 30.3 million people in the United States had diabetes in 2017 (about 9.4% of the US population). Among them, 90–95% of the cases reported was type-2 diabetes [124]. The major factors contributing to type-2 diabetes are an unhealthy diet rich in refined grains, red and processed meat, and added-sugar-containing beverages [125,126]. There is no cure for diabetes yet [127], and the uses of medications can be costly and can cause adverse side-effects [11]. Medical professionals recommend a lifestyle modification with a healthy diet, along with regular physical activity to prevent or to mitigate the risks associated with type-2 diabetes [127]. Many studies show the potential of OBG to reduce postprandial glucose level. Based on scientific findings, the European Commission approved a health claim about the potentiality of OBG in reducing postprandial glycemia, for which one has to consume 4 g of OBG for each 30 g of available carbohydrates per meal [128], and this claim was supported by the findings of Granfeldt, Nyberg, and Björck (2008) [45].

OBG influences the glycemic response by retarding the digestion of starch [39,129]. OBG changes the microstructure of food products and reduces starch gelatinization, which, in turn, slows down starch digestibility. There is also a strong correlation between peak blood glucose, OBG content, and MW weight in the food consumed [44]. Since OBG is a high MW polysaccharide, it exhibits high viscosity even at a low concentration. The consumption of this soluble fiber increases the viscosity of the meal bolus in the stomach, which slows down the access of the digestive transit [2,130,131]. The increase in viscosity decreases the absorption and diffusion of glucose [40,41], which helps to reduce the postprandial hyperglycemia and insulin secretion [132]. OBG, along other fiber components of the cell walls, may together increase the fecal volume and weight. This is called the bulking effect. This effect improves the consistency of the stool and increases the frequency of defecation, eases gastric emptying, and prevents an upset stomach [47]. A reduction in the viscosity of oat gum (extracts from oats mainly consisting of OBG) with acid hydrolysis reduced its capacity to decrease the plasma glucose level and insulin response [46].

The effectiveness of OBG also depends on the cooking time, amount, duration of consumption, processing techniques, physiochemical properties, and the form of the food product [2,133]. Steel cut and large flake oats have a low glycemic index (GI) value, muesli and granola have medium GI values, whereas quick cooking oats, instant oats, and oat milk have higher GI values [2].

### 1.3. Oats and Obesity

According to the WHO, obesity or overweight is one of the leading health risk factors that account for 2.8 million of global mortality [134]. It further increases the risk of other non-communicable health complications like CVD, musculoskeletal disorders, diabetes, and cancers [135]. Studies show that the intake of dietary fiber and whole grain mediate for body weight loss, and this would be a new avenue toward combating current obesity trends. The consumption of oat products with high OBG gives a perception of satiety and stomach distention, which reduces hunger [47,52]. Because of the viscous and hydration property of OBG, it delays gastric emptying, which inhibits the food intake, thereby reducing the overall body weight, body fat, body mass index (BMI), and central adiposity [47,48,136]. In addition, OBG also activates the gut hypothalamic axis, which increases satiety [12,49].

Studies on animal models show the effects of oats in the modulation of gut microbiota and its subsequent effect on reducing obesity. An oat-supplemented diet on rats decreased the body weight, epididymal fat accumulation, serum glucose, and serum lipid levels, and improved the composition of beneficial gut microbes, thus reducing metabolic-related disorders [51].

### 1.4. Oats and Celiac Disease

Celiac disease (CD), also known as gluten intolerance, is a systemic immune mediated gastrointestinal disorder induced by the ingestion of dietary gluten or related proteins in genetically susceptible people [137]. Gluten is a storage protein found in grains such as wheat, barley, rye, and similar grains enriched with glutamine and proline, which cannot be properly digested by the upper gastrointestinal tract and can damage the small-intestinal mucosa [138]. Adherence to a gluten-free diet seems to be the best therapy to treat CD [139]. The European Commission Regulation No. 41/2009, of 20 January 2009 [140], reports that oats can be included in the diet of people with gluten allergy. However, oats being used in a gluten-free diet should be produced, prepared, and/or processed with the care to prevent contamination by other gluten-containing grains, like wheat, rye, and barley [121,140]. Besides this, studies have shown that the immunogenicity of oats also depends on the varieties, which opens up new avenues for a thorough study of oat varieties before it is chosen as an ingredient to make gluten-free food products [141]. It is suggested that oats should be added to gluten-free adult diet only after CD symptoms like weight loss disappeared and growth parameters were maintained, after 6 months of a conventional gluten-free diet, and also after serological parameters get normalized, because there is a potential for sensitivity in oats [56]. Janatuinen et al. (1995) [53] compared diets with and without oats in adult CD patients. The majority of adult CD patients who had a mean intake of 49.9 ± 14.7 g per day of oats or oat products for 6 months and 46.6 ± 13.3 g per day for 12 months did not have unfavorable effects with the ingestion of a moderate amount of oats in their diet. Hoffenberg et al. (2000) [54] suggested that the consumption of oat cereals is safe for children newly diagnosed with CD with a 6-month study on 10 children, providing them with 24 g of oat at breakfast every day. Health Canada and the Canadian Celiac association also concluded that the ingestion of a moderate amount of pure oats can be tolerated by CD patients [142]. Different reviews and meta-analysis lead to the same conclusion; the consumption of a moderate amount of oats was associated with no effects on the symptoms, histology, immunity, or serological features of CD patients [143,144,145]. A study evaluated the long-term consumption of an oat-based diet by celiac patients and reported no small-bowel mucosal villous damage, inflammation, or gastrointestinal symptoms, with the consumption of an average of 24 g of oat-based diet daily for 8 years [55]. However, Schmitz (1997) [57] states that consuming large amounts of oats (100–160 g) daily over a long period of time might be toxic for patients with CD.

Wheat, barley, and rye are grasses from the Triticeae family and possess proteins (gliadin, secalin, and hordein) that are toxic to CD. These toxic proteins are called prolamins because they are rich in proline and glutamine. Oats belong to the Aveneae family and have structures of seed-proteins different from those of the grains from the Triticeae family [146]. Avenins, the prolamin of oats, have a lower proline content and a chemical structure similar to that of the gluten proteins. They have a smaller number of toxic sequences per unit weight of oats [141]. The tetrapeptide motifs -Gln-Gln-Gln-Pro-, -Pro-Ser-Gln-Gln-, -Gln-Gln-Pro-Tyr, -Gln-Tyr-Pro-Tyr- are the sequences characteristic of toxic gliadin [147,148]. Avenins are present in smaller amounts as a proportion of oat seed proteins in comparison to gluten proteins in wheat, rye, and barley. Hence, the doses of oat that are commonly consumed are safe [149,150].

Individual dietary prescriptions and an appropriate use through histologic and serologic studies are important to prevent unnecessary dietary restrictions and medical complications [151]. Still, it is recommended to include oats in the diet of CD patients, because it can increase the diversity in diets for CD patients and provide a good source of dietary fiber, vitamins, and minerals [152].

### 1.5. Oats and Cancer

Several studies have pointed out the positive association of biologically active compounds in oats and a reduction in cancer [12]. Antitumor activity and cancer prevention of OBG were observed against skin cancer cells [58], epithelial lung cancer [59], and colon carcinoma [60]. It was found that OBG was cytotoxic and induced oxidative stress in lung cancer cells compared to the normal cells [59]. Both soluble and insoluble beta-glucan were proven to reduce fecal bile acid levels, produce SCFAs, and help paracancerous apoptosis in mice, which ultimately helped to prevent colon carcinoma [60]. Attenuating effects of avenanthramides, a unique phytochemical present in oats, have been observed in an in vitro study where the results suggested that the consumption of oats and oat bran could reduce the proliferation of colon cancer cells [61,153]. Similarly, steroidal saponins avenacoside A and avenacoside B, defensive compounds present in plants including oats, are reported to inhibit the growth of human colon cancer cells in an in vitro model. However, both types of avenacosides showed weak inhibitory effects against the growth of the human colon cell lines that were tested (HCT-116 and HT-29) [20]. However, this study has opened an avenue for further study on the prospects of avenacosides as chemopreventive agents for other types of cancer [12]. A systematic review from 2014 concluded that oats or oat bran might have some preventive effects on colorectal adenoma and cancer [154].

### 1.6. Oats for Immunomodulation

The term immunomodulation refers to the process of bringing immune response to desired level in humans. OBG stimulates the production of interleukin-1 (IL-1) cytokines from murine peritoneal macrophages and tumor necrosis factor alpha (TNF-α) cytokines from a murine macrophage cell line in vitro [70]. These cytokines result in the induction of adaptive immune responses. In vitro studies of human dendritic cells, small intestinal cell lines, and colon cell lines also showed the immunomodulatory effect of OBG [65,68].

Immune modulating properties of OBG were observed in an in vivo study when OBG was given intragastrically or parenterally to mice infected with *Eimeria vermiformis* [63,64], and in another study where OBG enhanced the activity of transcription factors in intestinal leukocytes and enterocytes in mice [66]. The resistance to bacterial infection on mice challenged with *Staphylococcus aureus* was enhanced by a single intraperitoneal administration of OBG before the bacterial challenge [62]. A combination of moderate exercise and OBG in a diet showed an increase in the immune function and in the resistance of macrophages against herpes simplex virus type 1 (HSV-1) in mice [67].

### 1.7. Oats for Antioxidant Activity

Oxidation is a chemical reaction which produces free radicals. Free radicals damage the cells of organisms. Antioxidants are the compound that scavenges these free radicals, thereby reducing oxidative stress. Oat is rich in antioxidants such as vitamin E, phytic acid, phenolic compounds, flavonoids, sterols, and avenanthramides. Vitamin E (tocols) is an important antioxidant that is credited for preventing premature aging, chronic disease, cancer, CVDs, and strokes [17,155].

An in vivo study showed that the supplementation of high and low MW OBG effectively reduces the oxidative stress in mice or rats with lipopolysaccharide (LPS)-induced enteritis. A high MW OBG creates an environment for the regeneration of mucosal membrane, thereby reducing oxidative stress [69]. Oat vinegar showed antioxidant activity in vitro as well as in vivo through stronger radical scavenging activities and the inhibition of lipid peroxidation [73].

Avenanthramides (AVAs) are the most abundant phenolic alkaloids, found exclusively in oats, among cereals. More than 25 AVA compounds in oats have been reported, and the most abundant AVAs in oats are 2C, 2F, and 2P, with AVA 2C having the highest total antioxidant capacity [156]. The antioxidant activity of AVA is mainly through its ability to trap reactive oxygen species (ROS) [156], both hydroxyl groups, and α,β-unsaturated carbonyl moiety, which act directly or indirectly, are crucial for its antioxidant properties [3].

The intake of AVA increases the antioxidant defense in vivo, increasing the level of glutathione and significantly decreasing malondialdehyde [16,74]. The antioxidant activity of AVAs was also observed in vitro, where the AVAs inhibited the activity of β-carotene bleaching and the reaction with the free radical 2,2-diphenyl-1-picrylhydrazyl (DPPH) [71]. A novel mechanism of antioxidant activity of AVAs were shown in human kidney cells where the antioxidant activity was observed by the expression of a mediating factor called heme oxygenase-1 (HO-1) through the activation of nuclear factor -E2-related factor 2 (Nrf2) [72].

### 1.8. Oats and Gut Microbiota

Gut microbiota are important for proper gastrointestinal health and help prevent many diseases, like obesity, diabetes, and colorectal cancer. Whole oat possesses unique phytochemicals, including high beta-glucan, high lipid, and phenolics, which play an important role in gut health [83,84,85].

The in vitro fermentation of OBG produced SCFA, mostly propionate. The produced SCFAs are either absorbed back into the circulation, thereby affecting the regulation of metabolism, or utilized by other microbes, inhibiting harmful enzymes in the intestine [76,77,157,158,159]. The intake of OBG in rats resulted in an increase in fecal water content and lower pH, ammonia levels, β-glucuronidase activity, and colonic SCFAs [77], all associated with improved gut health. Similar results were obtained in a clinical study where subjects fed with OBG showed an increase in total SCFAs (particularly butyrate) [78,113].

Changes in gut microbiota from the supplementation with oat products helped to attenuate obesity and other obesity-related metabolic disorders in obese rats, as well as maintain the level of cholesterol, triacylglycerol, endotoxin, mRNA expression, and tumor cell necrosis factor –α [50]. An increased SCFA production and modulation of gut microbiota was also observed in hamsters when fed with oat and tartary buckwheat-based food [80]. Oat bran promoted the growth of cellulolytic bacteria in the hindgut of growing pigs [81] and showed bifidogenic effects in an in vitro model of gut microbiota [82].

In addition, resistant starch (RS), one of the functional dietary fibers from oats, has also shown beneficial effects on gut health. An in vitro study showed the gut microbiota modulation abilities of different sizes of oat flakes. In this study, the thick size of oat flakes showed a bifidogenic effect and an increase in butyrate production compared to the thin flakes because of the high RS in thick oat flakes [79]. Avenanthramides from whole oats can be effectively utilized by the gut microbiota, produce bioactive metabolites, and inhibit obesity by regulating the intestinal microflora and reducing the growth of harmful microbes [13,86,160].

### 1.9. Oats for Inflammation

The anti-inflammatory properties of oats have been observed in many studies. An aqueous extract of beta-glucan from oats, both high and low MW, showed anti-inflammatory effects in chronic LPS-induced enteritis [69,87]. In an in vivo study, purified OBG provided against acute gastritis decreased the strong activity of inflammation markers, TNF-α, and IL-10 [70].

OBG exhibited a protective effect against ulcerative colitis, which was induced by dextran-sulfate sodium (DSS). OBG reduced the disease activity index and colon tissue damage [88]. OBG also proved to be effective in the reduction of lipid peroxidation and inflammation caused by exercise [161]. In addition, a study found that oat protein supplementation can help alleviate the side effects of eccentric exercise like induced muscle damage, skeletal soreness, and loss of performance after downhill running or vertical jump [89]. AVA supplementation in women showed a significant decrease of neutrophil respiratory burst, NFκB activation, plasma IL-6 concentration, erythrocyte glutathione peroxidase activity, and increase of glutathione levels, which suggests that AVAs are a promising supplementation to decrease the systemic inflammatory response and attenuate the inflammation triggered by high physical exercise [90,91]. Another study also suggested that AVAs are potential inhibitors of the NFκB-mediated inflammatory response in select cell lines [92]. Avenanthramide C (AVC) from germinated oats were suggested as potential therapeutics for mast-cell-mediated allergic inflammation through the release of inflammatory mediators, histamine, and pro-inflammatory cytokines [93].

### 1.10. Oats for Atherosclerosis

Atherosclerosis is a disease where fatty materials called plaque are deposited on the inner walls of arteries. The proliferation of smooth muscle cells (SMC) and impaired nitric oxide (NO) production are two crucial steps in the development of atherosclerosis. Many studies have indicated the beneficial roles of oats on the prevention of atherosclerosis. In an in vitro study, synthetically prepared AVA significantly inhibited the serum-induced SMC proliferation and increased the NO production in both SMCs and human aortic endothelial cells (HAECs) [94]. Another in vivo study showed a reduction in atherosclerosis after the simultaneous intake of oat bran and atorvastatin (a drug used with diet to reduce bad cholesterol and fat) [162]. Oat-based diets significantly reduced the high-fat-diet-induced atheroma lesions in the aortic valve of Ldlr−/− mice [95]. One of the recent findings showed the anti-atherosclerotic potential of oat fiber through the activation of the SREBPs/LXRα pathway and by improving lipid metabolism [38].

### 1.11. Oats as Antimicrobial

An in vivo study where the mice were immunosuppressed and infected with oocytes *Eimeria vermiformis* showed reduced fecal oocyst shedding, no mortality, and a higher amount of immunoglobulin in the serum, suggesting the antimicrobial activity of OBG through the resistance to *E. vermiformis* infection [97]. Similar results were obtained in another study from the same author, where OBG treatment improved the resistance to *Staphylococcus aureus* and *E. vermiformis* [98]. Oat bran pretreated with viscozyme and cellulase significantly inhibited the growth of *Escherichia coli*, and oat bran pretreated with viscozyme and α-amylase enhanced the growth of *Bacillus subtilis* (a non-pathogenic bacterium) [99]. The saponin compound avenacin, from oat seeding roots, has been found to have an antifungal property and to inhibit various soilborne pathogens [163].

### 1.12. Oats for Dermatological Disorders

OBG has been well known for soothing, moisturizing, and irritation prevention properties; hence, it is used in many cosmetics and personal care products [164]. An ex vivo and in vivo study showed that 0.5% of OBG solution with a dose of 5 mg per cm^2^ deeply penetrated the skin into the epidermis and dermis and significantly reduced the wrinkle depth and height and the roughness of the skin [100]. An assessor-blind clinical trial conducted on acute burn patients showed that the patients experienced less itching and requested significantly less antihistamine when using liquid paraffin with 5% colloidal oatmeal compared to liquid paraffin only [101]. Colloidal oatmeal has also been found to be beneficial in the treatment of psoriasis and atopic dermatitis [165,166,167]. One of the studies showed the effective treatment of acneiform eruption induced by an epidermal growth factor receptor and multiple tyrosine kinase inhibitors via a colloidal oatmeal lotion [102]. Besides, colloidal oatmeal has been used in the treatment of a viral infection called *Molluscum contagiosum*, caused by poxvirus [103]. In addition, colloidal oatmeal is used for skin protection against ultra-violet (UV) light, as flavonoids in oats absorb the UV light in the range of 320 to 370 nm [168].

Many of the skin disorder treatments with oats or colloidal oatmeal are attributed to AVAs in oats. Studies have suggested that AVAs can inhibit the activity of nuclear factor-κB and the release of cytokines and histamines, causal reasons of inflammatory dermatoses [169]. AVAs modulate nerve responses and treat various dermatological disorders. They also prevent the chances of secondary inflammation disrupted barrier functions like atopic dermatitis and eczema by controlling the nerve responsible for itching [3,170]. Dihydroavenanthramide, a synthetic analog to the naturally occurring oat AVA showed the inhibitory effects on ultraviolet-B-induced ROS production and the expression of matrix metalloproteinases (MMPs) along with its molecular mechanism in human dermal fibroblasts [104].

## 2. Conclusions

Apart from pharmaceutical approaches, diet-based strategies are also documented to be effective against the prevention of different human diseases and mitigating disease risks. Oats can be presented as one of the most promising functional foods of the future, with many opportunities. Much evidence points to the beneficial effects of oats in the reduction of CVD risks, dermatologic disorders, inflammation, and type-2 diabetes, and these effects are mostly associated with OBG. Additional explorations of the beneficial effects of OBG on obesity, cancer, immune modulation, and gut health are needed, with more robust studies. Along with OBG, bioactive compounds in oat like phenolic acids, tocols, avenanthramides, and steroidal saponins have antioxidant properties and provide immense health benefits. However, only few studies have assessed the bioactivity of these compounds on human health. In addition, future studies should also consider other factors, such as the level of bioactive compounds, individual genetic differences, and the variations in the individual’s gut microbiota. Cultivar, processing methods and storage also causes differences in the amounts of bioactive compounds.

## Figures and Tables

**Table 1:** Use of oat/oat products and their health beneficial properties.

Beneficial Properties	Effect	References
Cardiovascular diseases	- Significant reduction on total cholesterol/LDL cholesterol/HDL cholesterol.	[22,23,24,25,26,27,28,29]
	- Cholesterol uptake by everted jejunal sacs inhibited by an increase in concentration of oat gum in mucosal mediums.	[30]
	- Fecal bile acids concentration significantly increased.	[26,31]
	- Significant decrease in serum cholesterol and cholic acid pool size. - Significant increase in deoxycholic acid pool size. - Significant increase in synthesis and fractional turnover rates of both primary bile acids. - No change in total bile acid pool size.	[32,33,34]
	- No significant effect of oat gum on serum total cholesterol, LDL cholesterol, and triglyceride concentrations showing the weak cholesterol-lowering capacity of oat gum, thereby suggesting that cholesterol-lowering is due to the solubility and viscosity of OBG and not to the concentration/amount.	[35]
	- Significant reduction in serum and liver cholesterol level with the consumption of high fiber oat flour containing 0–10% dietary fiber in hypercholesterolemic rats.	[25]
	- Increase in excretion of dry matter, fat, nitrogen, energy, and total bile acids; reduction in LDL and total cholesterol but no change in HDL cholesterol or lipo-proteins.	[36]
	- Significant reduction of serum cholesterol, peripheral serum SCFA levels increased.	[37]
	- Both total and LDL cholesterol decreased. - Activated cholesterol sensors LXRα and enhanced hepatic and intestinal cholesterol absorption.	[38]
Type II Diabetes	- Significant reduction of blood glucose and glycosylated serum proteins.	[39,40,41,42]
	- Reduction of postprandial plasma glucose and insulin concentrations.	[43]
	- Significant correlation between peak blood glucose and the product of extractable beta-glucan content and its MW.	[44]
	- Significant lower mean glycemic response and lower insulin response area (area under the curve) with oat bran concentrate bread. - The area under the curve for glucose and insulin was significantly lower, and the insulin peak was reached earlier with oat bran concentrate bread.	[42]
	- No significant difference in glycemic response fed with muesli with 3 g OBG, while a significant lowering of glycemic and insulin response was found with muesli with 4 g OBG compared to the referenced meal.	[45]
	- Reduction of viscosity of oat gum by acid hydrolysis decreased the postprandial glucose and insulin levels; oat gum’s ability to lower glycemic response was unchanged with the addition of maltodextrin.	[46]
Obesity	- Oatmeal suppressed appetite, increased satiety, and reduced energy intake.	[47]
	- Reduction of body weight, body fat, Body Mass Index (BMI), and waist to hip ratio.	[48,49]
	- Each oat product decreased body weight, epididymis fat accumulation, and serum lipid levels; improved metabolic disorder by enhancing gut microbial growth.	[50]
	- Effective reduction in body weight and fat and food efficiency but not appetite; reduction of serum glucose, free fatty acid, triacylglycerol (TG), total cholesterol, LDL, and HDL cholesterol; effectively reduced hepatic TG and cholesterol.	[51]
	- Diets with OBG significantly reduced the body weight of mice; the MW of OBG did not significantly affect the serum lipid profile.	[52]
Celiac Disease	- Patients on remission or newly diagnosed with celiac disease did not have worsening effects on duodenal villi or increased mononuclear cell infiltration.	[53]
	- Significant decrease in biopsy score, intraepithelial lymphocyte count, anti-tissue transglutaminase IgA antibody titer, and number of symptoms.	[54]
	- Consumption did not result in small-bowel mucosal villous damage, inflammation, or gastrointestinal symptoms.	[55]
	- All patients remained asymptomatic with normal levels of hematological and biochemical indices. - No change in endomysial and gliadin antibody values. - A standard histological evaluation showed no morphological damage. - No significant change in the quantitative histological examination.	[56]
	- A large consumption of oat products (100–160 g daily for a long time) may also contribute to the development of celiac disease.	[57]
Cancer	- Low MW OBG significantly decreased cancer cells’ viability, non-toxic for normal cells. - Showed a strong expression of caspase-12 in cancer cell lines.	[58]
	- OBG was cytotoxic and induced oxidative stress in cancer cells. - Human erythrocytes treated with OBG were less susceptible to hemolysis in a hypotonic solution.	[59]
	- The bile acid content was significantly reduced and the SCFA content was enhanced in both MW of OBG-administered mice; tumor cells apoptosis was significantly promoted.	[60]
	- Avenanthramide had no effect on cyclooxygenase-2 (COX-2) expression but inhibited COX enzyme activity and prostaglandin E2 production. - Significantly inhibited the cell proliferation of both COX-2-positive HT29, Caco-2, LS174T, and COX-2 negative HCT116 human colon cancer cell lines. - Had no effect on COX-2 expression and PGE2 production in Caco-2 and HT29 colon cancer cells.	[61]
	- Inhibitory effect of both Avenacoside A and B on human colon cancer cells HCT-116 and HT-29; Avenacoside B was more active than avenacoside A.	[20]
Immunomodulation	- In vitro stimulation of OBG resulted in the production of interleukin-1 (IL-1) in a dose- and time-dependent manner and only small amounts of tumor necrosis factor alpha (TNF-α). - Induced the production of IL-2, IFN-Y, and IL-4 in a dose-dependent manner in cultured spleen cells. - Survival of mice from *staphylococcus aureus* enhanced by intraperitoneal administration of 500 µg of OBG 3 days before bacterial exposure.	[62]
	- Parenteral administration of OBG resulted in a high level of total serum immunoglobulins and antigen-specific immunoglobulins. - Proliferative response to *E. vermiformis* significantly increased when OBG was provided 2 days before or at the time of infection.	[63]
	- Induced cytokines in bone-marrow-derived dendritic cells influencing immunomodulatory properties.	[64]
	- Digestion of OBG with endo-glucanase stimulated MCP-1, RANTES, IL-8, and IL-4 production in human dendritic cells, which resulted in the activation of receptor dectin-1.	[65]
	- Increased intestinal nuclear factor-κB (NF-κB) in leukocytes and enterocytes in the ileum but not in the colon of mice. - Level of interleukin-12 (IL-12) increased in intestinal lysates, whereas the concentration of interferon-Y decreased. - Tumor necrosis factor α showed reduced production in the colon.	[66]
	- Resistance of macrophage to Herpes Simplex Virus-1 increased with both exercise and OBG on the mouse model. Mortality also decreased.	[67]
	- OBG-enriched fecal water significantly increased IL-8 production in HT29 and INT407 cells. - Intercellular adhesion molecule (ICAM)-1 expression increased in T84 and caco-2 cells. - Antibody array showed an enhancement of inflammatory expression profiles.	[68]
Antioxidant activity	- Diet with high MW OBG decreased lipid superoxides, 7-ketosterol concentration, and GSSG activity in spleen.	[69]
	- Significant reduction of different type blood leucocytes - Higher reduction of lipid peroxidation observed with high MW OBG and low MW OBG led to a reduction in the enteritis group.	[70]
	- The elimination kinetics of plasma AVA followed first-order kinetics. - Bioavailability of AVA-A increased. - With the consumption of 1 g AVA enriched mixture, plasma reduced glutathione was increased.	[16]
	- Under the 2,2-diphenyl-1-picrylhydrazyl (DPPH) system, N-(3′,4′-dihydroxycinnamoyl)-5-hydroxyanthranilic acid (Bc) and N-(4′-hydroxy-3′-methoxycinnamoyl)-5-hydroxyanthranilic acid (Bf) were more active. - Bc had a higher activity than N-(4′-hydroxycinnamoyl)-5-hydroxyanthranilic acid (Bp) and Bf; Bc was as active as a standard synthetic antioxidant in the β-carotene system.	[71]
	- N-acetylcysteine attenuated AVA induced HO-1 (heme oxygenase-1), showing the role of reactive oxygen species; the hydrogenation of the double bond of the functional αβ-unsaturated carbonyl group of AVA removed their effects on HO-1 expression, which suggests that this group is essential for the antioxidant activity.	[72]
	- Decrease in malondialdehyde value, increase in activities of superoxide dismutase and glutathione peroxidase. - Hepatic damage induced by ^60^Co γ-irradiation was ameliorated in aged mice.	[73]
	- Significant increase in the level of Superoxide dismutase (SOD) and reduced glutathione hormone (GSH). - Significant decrease in malondialdehyde level.	[74]
Gut Health	- Significant increase in *C. histolyticum* subgroup in all vessels, and clostridial cluster IX maintained high populations with all fractions; propionate rich SCFA was observed.	[75]
	- Fermentation rate of oat was lower than that of wheat and rye bran. - Enzymatic digestion before in vitro colon fermentation retarded the ferment ability of oat samples. - Highest production of propionate from oats compared to wheat and rye.	[76]
	- Significant influence on fecal water content, pH value, ammonia levels, β-glucuronidase activity, azoreductase activity, and colonic SCFA concentrations. - Number of *Lactobacillus* and *Bifidobacterium* increased, but *Enterobacteriaceae* decreased in a dose-dependent manner.	[77]
	- Significant increase in the concentration of acetic, propionic, butyric, isobutyric, and isovaleric acid, but decrease in lactic acid.	[78]
	- Significant changes in total bacterial population after 24-h incubation, except for small flakes. - Small flakes had a significant increase in the *Bacteroides-Prevotella* group. - Large flakes significantly increased *Bifidobacterium* and also resulted in propionate rich SCFA with a significant increase in butyrate.	[79]
	- Reduction in body weight, epididymal fat accumulation, serum inflammatory factor levels, and regulated serum lipid levels. - Shifted the overall structure of microbiota in obese rats- Abundance of *Bacteroides* and *Firmicutes* and their ratio altered toward normal rats and significantly increased SCFA concentration in colonic digestion.	[50]
	- Significant increase in acetate, propionate, butyrate, and total SCFA concentrations in hamsters fed with OF compared to placebo.	[80]
	- Abundance of *Prevotella*, *Butyricicoccus*, and *Catenibacterium* was higher, while *Coprococcus* and *Desulfovibrio* were lower in oat bran-based diet.	[81]
	- Significant increase of Proteobacteria at 10 h, Bacteroidetes at 24 h, and concentration of acetic and propionic acid increased at 10 h and 24 h compared to control diet; 1% oat bran fermentation resulted in an increase in SCFA production at 24 h. - Relative abundance of *Bifidobacterium* unassigned at 10 h and *Bifidobacterium adolescentis* at 10 and 24 h compared to control diet.	[82]
	- Reduction in fecal levels of β-galactosidase and urease, whereas colonic fermentation capacity, excretion of SCFA, and rectal inflammation assessed through PGE_2_ levels were not changed.	[83]
	- Relative abundance of *Bacteroidaceae, Bifidobacteriaceae, Lactobacillaceae, Prevotellaceae, Ruminococcaceae,* and *Veillonellaceae* increased, and *Enterobacteriaceae* decreased. - Increased production of SCFA with higher concentration of acetate.	[84]
	- Relative abundance of *Prevotellaceae, Lactobacillaceae,* and *Alcaligenaceae* families in whole grain oat flour was higher, and the *Clostridiaceae* and *Lachnospiraceae* families were higher in low bran oat flour.	[85]
	- 8 different metabolites found in 2C treated mouse urine samples. - 2C was converted into 5-hydroxyanthranilic acid (M1), dihydrocaffeic acid (M2), caffeic acid, and M6 (dihydroavenanthramide-C); it also showed that 2C and its major metabolite M6 act against human colon cancer cells.	[86]
Inflammation	- Significant reduction of different types of blood leucocytes (lymphocytes T and B, granulocytes, and lymphocytes Tc) in rats with dietary high and low MW OBG.	[87]
	- Supplementation with OBG following LPS treatment reversed its effects; improvement in SCFA concentration. - Increase in the number of lactic acid bacteria.	[69]
	- High MW OBG decreased the tumor necrosis factor-α (TNF-α) in both experimental groups, while low MW OBG decreased the concentration of interleukin-10 (IL-10) in the gastritis group.	[70]
	- Significant reduction in clinical symptoms like less weight loss, diarrhea, and shortening of the colon. - Severity of colitis was significantly inhibited. - Decreased myeloperoxidase activity, nitric oxide, and malondialdehyde. - Inhibited mRNA and protein expression of pro-inflammatory factors like TNF-α, IL-1β, IL-6, and iNOS.	[88]
	- Oat proteins for 19 days reduced eccentric-exercise-induced skeletal muscle soreness, IL-6 concentration levels, myoglobin, and C reactive protein contents.	[89]
	- Significant increase in resting plasma glutathione (GSH) concentration, decreased glutathione disulfide response to downhill run, and lowered erythrocyte GSH peroxidase activity.	[90]
	- Decrease in downhill-walking-induced neutrophil respiratory burst at 24 h and C-reactive proteins level at 48 h. - Plasma interleukin (IL)-1β and mononuclear cell nuclear factor binding were suppressed in the AVA group. - Erythrocyte superoxide dismutase activity increased.	[91]
	- Reduction of IκB ^4^ β (IKKβ) kinase activity in response to (tBHP) stimulation and suppressed tBHP-induced TNFα and IL-1β mRNA expression. - Increase in cyclooxygenase-2 (COX-2) proteins and luciferase activity with tBHP treatment reduced by 50%.	[92]
	- Inhibition of immunoglobulin (Ig)E-stimulated mast cells degranulation through suppression of phosphorylation of phosphoinositide 3-kinase and phospholipase Cγ1 and by decreasing calcium levels. - Inhibition of inflammatory cytokines secretion by suppressing signaling proteins Lyn, Syk, Akt, and nuclear factor-κB.	[93]
Atherosclerosis	- Reduction of atherosclerotic lesion with intake of atorvastatin and oat bran simultaneously.	[94]
	- High-fat-diet-induced atheroma lesions in aortic valve were reduced by both types of oat-based diets, but high fat containing regular oat brans with high levels of AVAs diet was more effective compared to high fat containing regular oat brans with low levels of AVAs diet.	[95]
	- Reduction of plasma cholesterol. - Lowered plasma triglycerides and increased fecal excretion of cholesterol and bile acids. - Reduced atherosclerotic lesion area in the descending aorta and aortic root. - Significant reduction of plasma levels of fibrinogen and soluble vascular cell adhesion molecule-1.	[96]
	- OBG treated groups had minimal clinical signs and no mortality compared to immunosuppressed mice. - Total IgG, IgG1, IgG2A, IgM, and IgA; specific IgG anti-sporotize and merozoite immunoglobulins in serum were significantly higher. - IFN-y- and IL-4-secreting cells were detected in spleen and mesenteric lymph nodes.	[97]
Antimicrobial	- Significant protection against *S. aureus* and reduction in fecal oocyst shedding infected with *E. vermiformis*. - Patency period was shorter and antigen-specific antibodies were significantly higher. - Number of IFN-y-secreting cells in the spleen increased. - Effective changes in the lymphocytes population in the mesenteric lymph nodes and Peyer’s patches in mice infected with *E. vermiformis.* - In vitro study showed an enhancement in phagocytic activity by OBG.	[98]
	- Oat brans pre-treated with viscozyme and cellulase inhibited the growth of *E. coli.* - Viscozyme and alpha amylase treated brans supported the growth of *Bacillus subtilis*, which is used as a pro-biotic bacterium.	[99]
	- Significant reduction of wrinkle depth, height, and overall roughness.	[100]
Dermatological disorder	- The group using the product with colloidal oatmeal reported significantly less itching and registered fewer requests for antihistamines.	[101]
	- No associated toxicities.	[102]
	- Response to the treatment of *Molluscum contagiosum* with zinc oxide cream with colloidal oatmeal.	[103]
	- Western blot and real-time PCR analysis showed that ultraviolet B (UVB) radiation induced the expression of matrix metalloproteinase (MMP) -1 and MMP-3 and blocked UVB-induced reactive oxygen species (ROS). - UVB-irradiated MMP expression regulation by inhibition of ROS-mediated MAPK/NF-jB and AP-1 activation.	[104]
